# Assessment Wine Aroma Persistence by Using an in Vivo PTR-ToF-MS Approach and Its Relationship with Salivary Parameters

**DOI:** 10.3390/molecules24071277

**Published:** 2019-04-02

**Authors:** Carolina Muñoz-González, Francis Canon, Gilles Feron, Elisabeth Guichard, Maria Angeles Pozo-Bayón

**Affiliations:** 1 Instituto de Investigación en Ciencias de la Alimentación (CIAL), Campus de Excelencia Científica, Consejo Superior de Investigaciones Científicas-Universidad Autónoma de Madrid (CSIC-UAM), 28049 Madrid, Spain; c.munoz@csic.es; 2 Centre des Sciences du Goût et de l’Alimentation, UMR1324 INRA, UMR6265 CNRS Université de Bourgogne, Agrosup Dijon, F-21000 Dijon, France; francis.canon@inra.fr (F.C.); gilles.feron@dijon.inra.fr (G.F.); elisabeth.guichard@inra.fr (E.G.)

**Keywords:** PTR-ToF-MS, wine aroma persistence, in vivo aroma release, commercial tannin extracts, saliva, interindividual differences

## Abstract

To better understand wine aroma persistence, the nasal cavity of nine volunteers was monitored by Proton Transfer Reaction-Time of Flight-Mass Spectrometry (PTR-ToF-MS) after they rinsed their mouths with three rosé wines (one control and the same wine supplemented with two tannin extracts) during four minutes. Wines were aromatised with a mixture of five target aroma compounds. Results showed that wine aroma persistence was highly compound-dependent: while esters disappeared very fast, other compounds such as linalool remained in the oral cavity for longer times after wine expectoration. A low effect of tannins (at 50 mg/L) on nasal cavity parameters was observed, with the exception for the compound ethyl decanoate that was significantly higher released in the presence of tannins. Strong interindividual differences on aroma persistence were also found. Significant positive correlations with the salivary total protein content and negative with the salivary flow were observed for specific compounds. This work has studied for the first time in vivo wine aroma persistence in real time from an analytical perspective.

## 1. Introduction

The hedonic nature of wine consumption is associated to the pleasant sensory experience that provides to the consumers in terms of colour, taste, mouthfeel, odour or aroma. Its perception begins in the wine glass, continues during wine ingestion and it can last once the wine is no longer in the mouth, sensation known under the term of “wine finish” [1]. One crucial aspect relative to the wine finish is the length of time that aroma lingers in the mouth. This has been related to diverse facts such as (i) the different adsorptive capacity of the aroma compounds to salivary proteins or to the oral or pharyngeal mucosa that could form oral/throat coatings containing aroma reservoirs [2,3] which, in turn, might be influenced by the wine matrix composition [4,5,6] and/or (ii) to the susceptibility of some aroma compounds or aroma precursors to be metabolised by components present in saliva [7,8,9,10,11].

Most of the studies about wine aroma persistence have been conducted using sensory dynamic approaches like time-intensity evaluation [12,13] or Temporal Dominance of Sensations [6,14]. These works have revealed that the type of volatile compound, the coexistence of different volatile compounds in the wine, or the presence of other matrix components, such as ethanol, or tannins might affect the intensity and length of aroma persistence. Other works have employed in vivo analytical approaches, such as the intra oral-SPME-GC/MS methodology [3,15] or a combination of analytical and sensory techniques [2,16], in order to elucidate the chemical reactions behind the sensory results. However, none of the in vivo analytical works carried out on this topic have studied the kinetic of release in real time. To do that, the use of direct injection mass spectrometry, such as atmospheric pressure chemical ionization (APCI) systems or proton transfer reaction mass spectrometry (PTR-MS) is required. Nevertheless, the measurement of ethanolic solutions by these techniques present technical difficulties since changing the ethanol concentration in the source made reliable measurements difficult. Very recently, a new methodology has been developed by Semon and collaborators [17] in which the parameters of the PTR/MS instrument has been adjusted to allow ethanol chemical ionization conditions while minimizing the protonated molecule fragmentation of the volatiles with no compromise in sensitivity for in vivo applications. This methodology has been successfully employed by Arvisenet and collaborators [18] to evaluate the effect of sugar and acid composition on in vivo aroma release in model wines. However, wine is a complex matrix formed by many non-volatile components that might affect aroma release and persistence. In fact, the presence of phenolic compounds has been recently proposed as a factor that could modulate wine aroma release in the mouth [15,16].

Therefore, the objective of the work was to better understand the behaviour of aroma compounds in the mouth under real consumption conditions and using real wines. To do that, nine volunteers rinsed their mouths with three wines flavoured with a mixture of five target aromas. To identify the possible matrix effects, the wines were a rosé wine (control wine) and the same rosé wine supplemented with two commercial tannin extracts at 50 mg/L. The aroma release over 4 min after wine expectoration was followed in real time by PTR-ToF-MS. Finally, nasal cavity data were correlated with salivary parameters in order to elucidate the role of saliva on wine aroma persistence.

## 2. Results and Discussion

### 2.1. Wine Chemical Composition

The chemical characterization of the three wines employed in this study was performed and showed in Table 1 together with the results of the ANOVA and LSD test. As it can be seen, the three wines exhibited significant differences in their composition in terms of pH, colour intensity, total polyphenols and total procyanidins. As expected, the wines supplemented with the commercial tannin extracts were richer in total polyphenols than the CW. In addition, PW that was supplemented with a procyanidin rich extract was the wine with the highest procyanidin content.

### 2.2. Influence of Aroma Compound Type on Oral Aroma Persistence by PTR-ToF-MS

**Figure 1:**
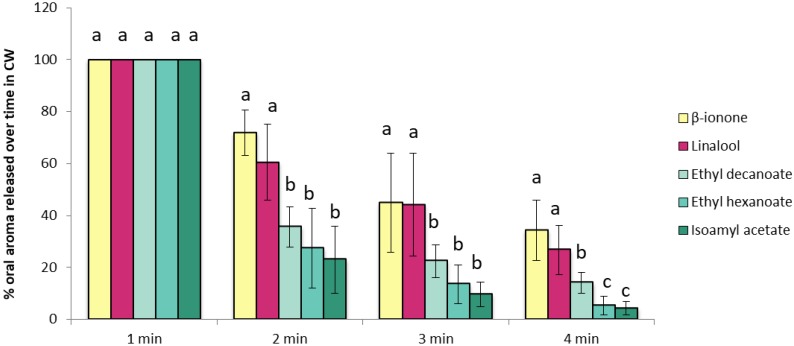
Percentage of aroma released over time (AUC values) obtained for the volunteers (*n* = 9) after rinsing their mouths with the CW. Different letters across the different compounds denote statistical differences after the application of Tukey test.

The analysis of the oral aroma persistence was done monitoring the nasal cavity of nine subjects by PTR-ToF-MS during 4 min after they rinsed their mouths with the CW. Figure 1 shows these results. Data were normalised considering the first minute after wine expectoration as the 100% and calculating the percentage relative to the first minute for the following four minutes to allow compound comparison. Data represent the mean values for all the subjects (*n* = 9).

As expected, once the wine disappeared from the oral cavity there was a progressive oral aroma decrease (Figure 1). However, the extent of the decrease was compound-dependent and the compounds did not disappear at the same rate in the oral cavity. As can be seen, the three esters disappeared significantly faster than the terpene-alcohol and the C_13_-norisoprenoid. In fact, four minutes after expectoration these compounds (linalool, β-ionone) were still present in the mouth at a 40% regarding the first minute after spitting out the wine; while others (isoamyl acetate, ethyl hexanoate) had almost disappeared at that time. The differences observed in this study confirm the results obtained in a previous work performed in vivo by Esteban-Fernandez and collaborators [3] but with another analytical technique. In that study, an intraoral-SPME-GC/MS methodology was used to evaluate the impact of the chemical characteristics of aroma compounds on aroma persistence after exposing the oral cavity to a wine aromatized with six target aroma compounds (isoamyl acetate, ethyl hexanoate, linalool, guaiacol, β-phenylethyl ethanol and β-ionone). The authors observed that esters disappeared very rapidly from the oral cavity after wine rinsing but compounds such as linalool and β-ionone remained in the oral cavity for longer times after expectoration. Therefore, having these two studies in mind, it can be stated that after the consumption of wine, there is a progressive intra-oral aroma decrease at different decay rates depending on compound type. This implies that compounds such as linalool or β-ionone (with floral, violet notes) might contribute in an important way to wine aroma persistence than esters (fruity notes).

Interestingly, the compounds that were less persistent in the breath were the esters in both in vivo studies. Esters have been described to be submitted to metabolisation due to components present in the saliva [7,8,10,16,19]. Therefore, a transformation of these compounds in the oral cavity could explain the low persistence of esters after wine expectoration. In order to check this hypothesis, the *m*/*z* corresponding to the expected metabolites (acids) were extracted and the dynamic curves generated (data not shown). However, these *m*/*z* also corresponded to fragments of the esters formed during the ionization process, and thus this hypothesis could not be confirmed in the present study.

Metabolisation processes occurring in the oral cavity could not to be the main or unique mechanism explaining the effects of saliva on aroma compounds and other factors related to physicochemical properties of aroma compounds (Table 2) could drive oral aroma persistence. Non-covalent interactions of aroma compounds with salivary proteins have been proposed to be important for in-mouth processing [3]. These interactions could be mediated by physicochemical properties of the compounds such as their hydrophobicity. However, in this work, the most hydrophobic compound (ethyl decanoate log *P* = 4.79) was less persistent in the breath than other less hydrophobic (linalool log *P* = 2.97). This fact is no surprising since previous studies found that the impact of human saliva on aroma compounds is highly chemical family-dependent [7,8,10]. Having a look within the chemical family of esters, the most hydrophilic one (isoamyl acetate) was less persistent in the breath four minutes after spitting out the wine than the most hydrophobic ester (ethyl decanoate) (Figure 1). This suggests that apart from the possible enzymatic reactions other phenomenon such as non-covalent interactions with salivary proteins could also drive oral aroma persistence on esters.

### 2.3. Oral Aroma Persistence Depending on Individual and Wine Type

To study the influence of wine type and of individual on oral aroma persistence, the nasal cavity of nine volunteers was monitored by PTR-ToF-MS during four minutes after they rinsed their mouths with three different wines spiked or not with two oenological tannins (procyanidin and ellagic types). From the dynamic release curves of each aroma compound, different parameters described in the M&M section were extracted and submitted to two-way ANOVA analyses (Table S1).

**Figure 2:**
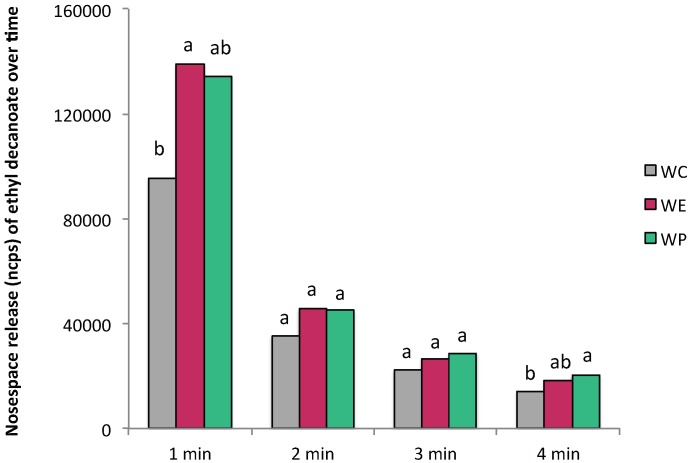
Mean values (ncps) of ethyl decanoate released in each of the wine types by PTR-ToF-MS. Data represent the AUC values determined in the subjects in each minute of analyses. Different letters denote statistical significant differences (*p* < 0.05) from Tukey test.

Results of the two-way ANOVA showed that the effect of wine type was not significant for most of the aroma compounds and nasal cavity parameters (Table S1). Only the compound ethyl decanoate was significantly affected by the presence of tannin extracts in terms of AUC (Table S1). As it can be observed in Figure 2, the presence of tannins (50 mg/L) for this compound was traduced in a higher release that was significant in the first and fourth minutes of nasal cavity monitoring compared to the CW. Different hypotheses could explain these results. Firstly, tannins are known to interact non-covalently and through mutual hydrophobicity with aroma compounds in wines [20]. It has also been proposed that tannins participate in the formation of large complexes (salivary proteins-wine tannins-wine carbohydrates) able to encapsulate hydrophobic aroma molecules that would interact with the mucosa remaining in the oral cavity for longer times [4,5,21]. Therefore, tannins could have promoted a higher retention of the aroma compounds (and especially of hydrophobic compounds) during wine rinsing that would be traduced in a higher release over time once the wine was expectorated. Secondly, the presence of tannins could have inhibited certain salivary enzymes implicated in the metabolism of aroma compounds, such as ethyl esters, in the mouth. These two hypotheses would explain the higher release of ethyl decanoate in the presence of tannins.

However, these results contradict a previous study [16] that showed a general intra-oral aroma decrease in the presence of tannins. Nevertheless, it is worth mentioning that in the present study the amount of tannins added was considerably smaller than in the study mentioned above (50 vs. 150 mg/L). Actually, previous in vitro studies have already shown that tannin concentration greatly influences the type of interactions with aroma compounds and their effect on aroma release [4,22].

For the other four aroma compounds, the two oenological tannin extracts did not have a significant effect on wine aroma persistence at the concentrations used (Table S1). However, the effects of tannins on aroma persistence at a sensory level will need to be validated in further experiments. In fact, it is possible that although there was no an effect of tannins on the real amount of aroma that reach the olfactory receptors over time, perceptual effects of taste or astringency can modify the perception of aroma and its duration [18].

**Figure 3:**
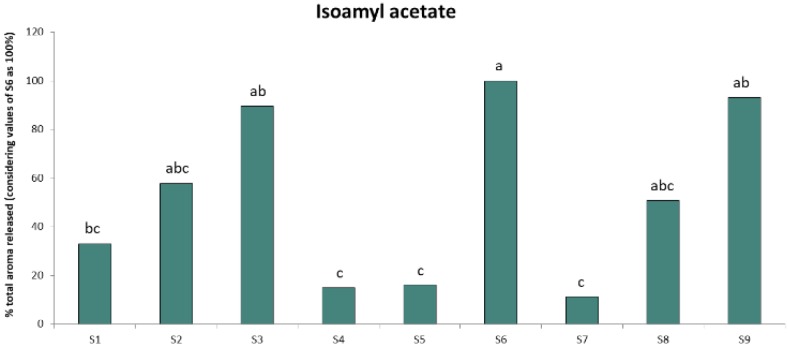
Mean values (%) of isoamyl acetate released for each of the volunteers by PTR-ToF-MS. Data represent the total AUC values for isoamyl acetate determined in the subjects and are expressed in percentage (considering the values obtained for the S6 as 100% and comparing this value with the amount determined for the rest of the subjects). Different letters denote statistical significant differences (*p* < 0.05) from Tukey test.

Regarding the effect of interindividual differences on nasal cavity data, two-way ANOVA showed that this factor was significant (*p* < 0.05) for almost all the parameters extracted in the five compounds of the study (Table S1). These results confirmed the high interindividual variability in oral aroma release [18,23] and persistence [3,15,16] previously found. The release profile obtained for the subjects was consistent for all the aroma compounds regarding the AUC values, which means that S6 was always the highest releaser while S7 the lowest for all the aroma compounds. An example of interindividual variation for the compound isoamyl acetate it is shown in Figure 3.

### 2.4. Relationship between Nasal Cavity and Salivary Parameters

Results discussed above indicated large interindividual differences in the nasal cavity parameters extracted from the release curves. Differences associated to the physiology of the subject, and specifically to the oral physiology of the volunteers could explain these results. Among the factors contributing to the oral processing of food, saliva has been described as one of the key factors in flavour perception [24,25]. During wine consumption, aroma compounds are transferred from wine to the saliva phase, then interact with the oral/pharyngeal cavity and/or are released via the airflow to the olfactory receptor. Thus, in these fast and complex processes, saliva governs the rate and amount of compounds that reach the olfactory receptors over time by controlling the transport of aroma molecules to their receptors, their adsorption onto the mouth surfaces (i.e., oral mucosa), their metabolism by, for instance, enzymatic modification, and their interaction with food matrix components such as tannins.

**Figure 4:**
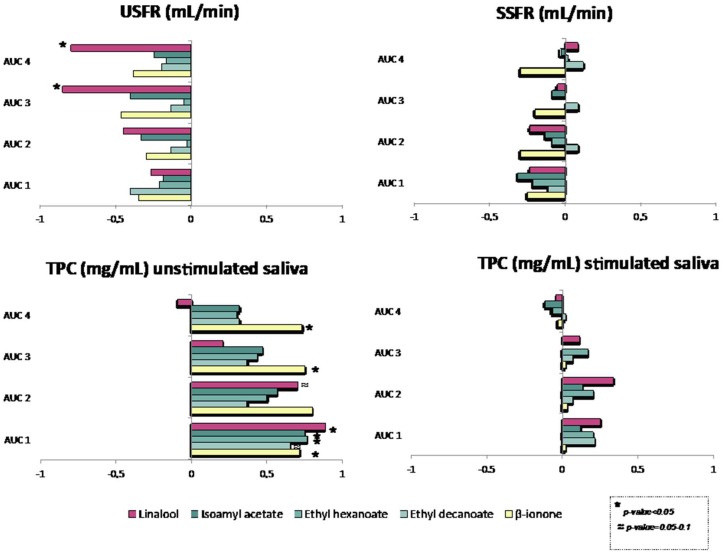
Spearman correlation matrix between salivary parameters and AUC data determined by PTR-ToF-MS.

In an attempt to investigate the relationship between saliva and aroma persistence, Spearman correlations between the nasal cavity data (AUC) and the salivary parameters determined from each of the subjects (unstimulated and stimulated salivary flow and total protein content in both types of salivas) were carried out (Figure 4). As it can be seen, a quick look of the figure indicates that unstimulated salivary parameters were more correlated to nasal cavity data than stimulated parameters. This could be due to the fact that wine is a liquid matrix that does not require an important degree of saliva stimulation during consumption.

Moreover, the salivary flow was negatively correlated with the AUC values for all the aroma compounds while TPC was positively correlated, which means that the lower the flow and the higher the protein content, the higher the AUC values over time. The correlations for USFR were significant in the case of linalool in the 3rd and 4th swallows and could be related to a cleaning effect of the salivary flow in the mouth. For TPC, the correlations were significant in the case of β-ionone in all the swallows, linalool in the 1st and 2nd swallows, and the three esters in the first swallow, and could be related either to a “salting out” effect or to a retention of aroma compounds by salivary proteins in the mouth. In general, these results seemed to indicate that salivary proteins could govern the release in the first moments after wine drinking whilst salivary flow could be more related to aroma persistence at longer times. However, a high number of subjects will be necessary to confirm these trends.

## 3. Materials and Methods

### 3.1. Aroma Compounds

Five typical wine aroma compounds were chosen for this investigation. These compounds included three esters (isoamyl acetate, ethyl hexanoate, ethyl decanoate), one terpene alcohol (linalool) and one C_13_-norisoprenoid (β-ionone). They presented different physicochemical characteristics (chemical family and chemical properties), and aroma descriptors (Table 2). The aroma compounds were of food-grade purchased from Sigma-Aldrich (Steinheim, Germany).

For the aromatisation of wines, five independent stock solutions were prepared in absolute ethanol. From those solutions, each aroma compound was added to the wines immediately prior to the experiments to obtain a final concentration of 0.0001 M. This concentration in an ethanolic environment allowed the complete solubility of aroma compounds [26]. Preliminary studies confirmed that the concentration selected allowed good sensitivity while avoiding instrument saturation. It was also taken into account that the aroma mixture was acceptable for the subjects who participated in the study.

### 3.2. Wine Samples

Three different wines were evaluated in this study. A commercial Cencibel rosé wine with an alcohol content of 12% was selected for its low aroma profile. This wine was considered the control wine of the study (CW). To obtain the two additional wines, the CW was spiked with two different commercial tannin extracts: (i) A commercial grape seed extract rich in procyanidins (Vitaflavan^®^ (DRT, Dax, France)) that led to a wine coded as PW, (ii) and a commercial extract of oak elagitannin (Samartan TT5^®^ Dolmar (La Rioja, Spain)) that led to the EW wine. The extracts were added to the CW at a concentration of 50 mg/L, stirred, aliquoted in dark bottles and kept refrigerated at 8 °C under a nitrogen atmosphere until analysis. This concentration was chosen following the recommendations of the producers for this type of oenological additives. In addition, this concentration allowed its complete solubility and did not induce changes in the astringency of wines.

### 3.3. Chemical Wine Matrix Composition

pH was determined using a pH meter (Mettler Toledo, Barcelona, Spain). Color intensity was calculated as the summation of absorbance measurements at wavelengths 420, 520 and 620 nm using a Specord^®^ plus (Analytik Jena AG, Jena, Germany) spectrophotometer. Protein content was determined by the standard Bradford protein assay using bovine serum albumin (BSA) as standard for calibration (Sigma-Aldrich, Lyon, France). The Folin–Ciocalteu method and spectrophotometric measurement at 670 nm [27] was used for total polyphenols determination. Total procyanidins were determined by the vanillin method following the procedure previously described [28].

### 3.4. In Vivo Nosespace Sessions

#### 3.4.1. Volunteers

Nine individuals (three men and six women) participated in the study. The participants were volunteers that participated for their motivation and availability to perform all the trials of the study. All of them were informed of the characteristics of the study and signed the informed consent document prior to their participation. The Bioethical Committee of the Spanish National Council of Research (CSIC) approved this study.

#### 3.4.2. Saliva Samples

##### Saliva Collection and Salivary Flow Rate Calculation

The subjects were requested not to eat, drink or smoke at least one hour before the collection of saliva samples. Unstimulated salivary flow was measured by instructing the subjects to let the saliva naturally be accumulated in the mouth and then spat out into a pre-weighed cup over a period of 10 min. For stimulated saliva, the subjects chewed a piece of Parafilm for a period of 5 min spitting out the saliva into a pre-weighed screw-cap cup every time they felt like swallowing. After that, the cups were weighted and the salivary flow rates calculated and expressed in mL/min assuming that 1 g of saliva corresponds to 1 mL. Immediately after collection, the saliva samples were stored at −80 °C until subjected to biochemical analyses.

##### Protein Concentration of Saliva Samples

Protein concentration was obtained by standard Bradford protein assay Quick Start (Bio-Rad, Roanne, France) using bovine serum albumin (Sigma-Aldrich) as standard for calibration.

### 3.5. Nosespace Analyses Using PTR-ToF-MS

Three sessions were established in which the volunteers evaluated in random order the CW (without tannin extract) and the wines with procyanidins (PW) and elagitannins (EW) extracts. The procedure consisted on monitoring the individual's nosespace after wine rinsing thanks to a Teflon nosespiece, that connected both nostrils of the subjects to a proton transfer reaction-mass spectrometer (PTR-MS) instrument equipped with a Time-of-Flight (ToF) analyser (PTR-ToF 8000, Ionicon Analytik, Innsbruck, Austria). The connection was ergonomic thanks to the use of a light helmet that enabled the subjects move their head freely. The helmet was connected to the transfer line of the PTR instrument by a flexible heated PEEK tubing and the sampling was performed at a total flow rate of 145 mL/min with the transfer line at 80 °C. Parameters of the PTR-MS were similar to those of previous studies [17,18] conducted on model wines which allowed ethanol chemical ionization conditions while minimizing the protonated molecule fragmentation of the volatiles. Briefly, the instrument drift tube was thermally controlled (80 °C) and operated with a voltage of 340 V and a pressure of 2.31 mbar resulting in an E/N ratio of 80 Td. Data acquisition was performed at 1 mass spectrum per 0.108 seconds ranging from *m*/*z* 0 to 250. Breath volatile concentrations were expressed as normalized cps, taking into account corrected transmission and normalization to the protonated water and the protonated ethanol (C_2_H_5_OH)H^+^ monitored at their respective ^18^O isotopic contributions found at *m*/*z* 21.022 (H_3_^18^O^+^) and *m*/*z* 49.054 (C_2_H_5_^18^OH_2_^+^). All the mass spectra were background-subtracted using the background signal measured (during 60 s) before sample introduction into the mouth.

After the monitoring of the breath noise, the subjects were instructed to put the wine sample (15 mL) into their mouth at one time. Once in the oral cavity, the participants were instructed to rinse their mouths with it during 30 s avoiding swallowing and without opening the soft palate. Effective closing of the velum was checked by PTR-MS signal. After this time, the volunteers were instructed to spit out the wine and then to swallow. Every 60 s, the subjects were instructed to swallow. In total, five swallows were performed, which corresponded to 4 min of monitoring after spitting out the sample. Between samples, the subjects were instructed to rinse their mouths with water, bicarbonate and pectin. The next sample was given to them 10 min after the cleaning procedure. All the wine samples were evaluated four times. The first sample evaluated was considered as a warm-up sample to familiarize subjects with protocol conditions.

**Figure 5:**
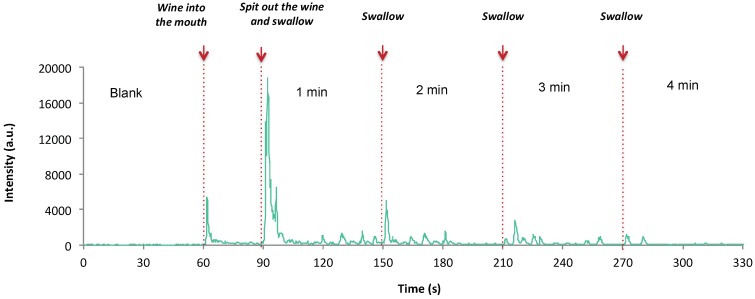
Example of oral aroma monitoring by PTR-ToF-MS.

An example of the release curve of one individual is given in Figure 5 for the ion 145.122 (ethyl hexanoate). All the release data were calculated from the breath concentration ncps data, using IGOR Pro (WaveMetrics, Inc. Portland, OR. USA). Several parameters were extracted from the release curves: maximal intensity for each minute (Imax), area under the curve (AUC) for each minute, % of release for each minute and time to reach the 25, 50, 75, 80, 90, 95% of total release (T25, T50, T75, T80, T90, T95), total release (total AUC) and slope of the total release.

### 3.6. Statistical Analyses

One-way ANOVA and the least significant differences (LSD) test were used to determine significant differences in the chemical composition of the three wine samples. Two-way ANOVA and the Tukey test were used to determine significant differences in nosespace parameters of the five aroma compounds considering individuals and wine type as factors. Spearman correlation analysis was applied to determine the existence of correlations between salivary parameters and AUC data in each sampling point for the five studied aroma compounds. The significance level was *p* < 05 throughout the study. The XL-Stat program was used for data processing (StatSoft, Inc., Tulsa, OK, USA, 2005, www.statsoft.com).

## 4. Conclusions

This work has studied for the first time in vivo wine aroma persistence using an analytical real time approach, therefore in closer conditions to wine consumption. Results from this work have shown that aroma persistence after oral wine exposure is compound-dependent. Esters disappeared very quickly from the oral cavity compared to compounds from other chemical families (terpene-alcohol, C_13_-norisoprenoid). This could be related to a metabolisis of esters in the oral cavity, which would contribute in a lesser extent to wine aroma persistence. Moreover, apart from metabolism, other mechanisms such as non-covalent interactions between aroma compounds and salivary proteins could govern the intra-oral release.

The addition of tannin extracts did not show a remarkable effect on aroma persistence, at the concentrations used, except for ethyl decanoate. However, to fully understand the effect of tannins on wine aroma persistence new multidisciplinary studies that combine in-mouth processes and sensory studies including multi-sensory integration in the brain are needed.

Important interindividual differences in oral aroma persistence have been observed in this study. Spearman correlations between nasal cavity and salivary parameters revealed that these correlations were positive for salivary proteins in the first moments after wine expectoration. This means that the higher the protein content the higher the release. This could be related to a “salting out” effect or to the retention of aroma compounds by salivary proteins in the mouth during wine rinsing, which could be further release in the followings swallowing episodes. Besides, negative correlations between salivary flow and nasal cavity parameters were found mostly at longer times after wine expectoration, which could be related to a cleaning effect by the replenishment of new saliva in the mouth. However, a large set of individuals will be needed to confirm these trends.

Finally, these findings could help to better understand interindividual differences in wine aroma persistence and could be considered by winemakers to improve the global quality of wines, and to make wines more adapted to specific consumers groups.

## Figures and Tables

**Table 1:** Chemical composition (mean ± SD) of the three wines employed in this study. The table also shows the ANOVA and LSD-test results to compare the statistical significance of wine matrix differences. Each sample was analysed in triplicate.

Wines	pH ± SD	CI ± SD	TPC (mg/L) ± SD	TP (mg Gallic Acid/L) ± SD	TPr (mg Catechin/L) ± SD
CW	3.07 ± 0.01 ^b^	0.94 ± 0.01 ^b^	5272.00 ± 249.80	239.92 ± 20.86 ^b^	111.21 ± 1.38 ^b^
EW	3.09 ± 0.00 ^a^	0.97 ± 0.01 ^a^	5697.33 ± 887.87	264.08 ± 8.45 ^ab^	110.16 ± 2.49 ^b^
PW	3.10 ± 0.01 ^a^	0.92 ± 0.01 ^c^	5740.67 ± 194.65	282.93 ± 2.18 ^a^	152.63 ± 4.59 ^a^
Anova Summary	pH *	CI *	TPC	TP *	TPr *
R^2^	0.918	0.880	0.181	0.732	0.979
F	33.500	22.076	0.661	8.185	482.872
Pr > F	0.001	0.002	0.550	0.019	<0.0001

SD: standard deviation; CI: colour intensity; TPC: total protein content; TP: total polyphenols; TPr: Total procyanidins. Different superscripts within the same column denote statistical differences (*p* < 0.05). Asterisk means a significant difference (*p* < 0.05) in this chemical parameter.

**Table 2:** Physicochemical characteristics and proton transfer reaction—time-of-flight—mass spectrometer fragmentation patterns of the aroma compounds added to the wines.

Compounds	CAS N°	Physicochemical Characteristics						Descriptor ^f^	Target Mass ^g^	
		Chemical Class ^a^	Chemical Formula	MM ^b^	*log P* ^c^	BP ^d^	Solubility ^e^		Ion	*m*/*z*
Isoamyl acetate	123-92-2	1	C_7_H_14_O_2_	130	2.25	135	2000	Banana	(C_7_H_14_O_2_)H^+^	131.1081
Ethyl hexanoate	123-66-0	1	C_8_H_16_O_2_	144	2.83	167	629	Apple	(C_8_H_16_O_2_)H^+^	145.1223
Ethyl decanoate	110-38-3	1	C_12_H_24_O_2_	200	4.79	248	4	Grape	(C_12_H_24_O_2_)H^+^	201.1776
Linalool	78-70-6	2	C_10_H_18_O	154	2.97	204	1590	Flower	(C_10_H_16_)H^+^	137.1325
β-ionone	8013-90-9	3	C_13_H_20_O	192	4.42	263	169	Violet	(C_13_H_20_O)H^+^	193.1592

^a^ 1: ester; 2: terpene alcohol; 3: C_13_ norisoprenoid. ^b^ Molecular mass (g/mol). ^c^ Hydrophobic constant estimated using molecular modeling software EPI Suite (US EPA 2000–2007). ^d^ Boiling point (ºC) estimated using molecular modeling software EPI Suite (US EPA 2000–2007). ^e^ Solubility (mg/L) estimated using molecular modeling software EPI Suite (US EPA 2000–2007). ^f^ From Flavornet (http://www.flavornet.org; accessed October 2009) database, from NIST web chemistry book (2005) (http://www.webbook.nis.gov/chemistry). ^g^ Masses monitored by PTR-ToF-MS.

## Supplementary Materials

The following are available online, Table S1: Results of the two-way ANOVA performed to compare the statistical significance of wine type and individual differences obtained in each of the nasal cavity parameters extracted from the in vivo release curves.
