# Supplementary material for: Assessment Wine Aroma Persistence by Using an in Vivo PTR-ToF-MS Approach and Its Relationship with Salivary Parameters
Source: Molecules. 2019 Apr 2;24(7):1277. doi: 10.3390/molecules24071277 (PMC6479722; doi:10.3390/molecules24071277)
Supplement: Supplementary file 1 [file molecules-24-01277-s001.pdf]

Table S1. Results of the two-way ANOVA performed to compare the statistical significance of wine type and individual differences obtained in each of the nasal cavity parameters extracted from the in vivo release curves.

		Wine type				Interindividual differences									
	**Time (min)**	**WC**	**WE**	**WP**	**p-value**	**S1**	**S2**	**S3**	**S4**	**S5**	**S6**	**S7**	**S8**	**S9**	**p-value**
**Isoamyl acetate**															
**Imax**	**1**	7260	10724	8268	0.311	4145	11028	16979	3016	1310	14286	2086	5129	20778	**0.001**
	**2**	1101	1386	1326	0.744	929	1307	1309	272	954	2178	242	1531	2721	0.059
	**3**	577	611	718	0.799	335	525	647	149	368	1292	124	926	1353	0.054
	**4**	232	312	317	0.458	174	185	590	71	111	520	84	307	539	**0.004**
**AUC**	**1**	186267	211798	210533	0.824	119092	230907	353566	62019	43325	411897	47142	169262	388585	**0.001**
	**2**	33504	34522	35062	0.971	27585	39391	53064	9005	25977	54698	5492	46622	47432	**0.005**
	**3**	15823	17486	21929	0.527	14175	17382	30270	4126	10960	30025	3163	28303	27308	0.069
	**4**	6919	9299	10844	0.343	7275	6736	19597	1971	4638	13061	1470	14779	10896	**0.018**
	**Total**	241593	273105	278368	0.772	168127	294416	456496	77121	82902	509681	57268	258966	474221	**0.001**
**% of release**	**1**	74	74	75	0.922	71	79	75	80	54	80	82	63	82	**0.002**
	**2**	17	15	14	0.364	17	13	13	12	31	11	10	20	10	**0.000**
	**3**	7	7	8	0.898	8	6	8	5	13	6	6	11	6	**0.038**
	**4**	3	4	4	0.658	4	2	4	3	5	3	3	6	2	0.066
**T25**		4	4	6	0.450	4	3	5	7	2	5	3	3	7	0.543
**T50**		15	14	15	0.977	22	8	18	13	5	29	10	9	19	0.131
**T75**		36	34	38	0.516	46	37	46	33	15	45	36	30	39	**0.016**
**T80**		41	40	46	0.506	60	42	52	39	21	48	38	39	44	**0.036**
**T90**		72	68	78	0.579	92	59	84	65	57	92	48	83	75	0.227
**T95**		108	107	122	0.445	135	99	135	96	97	136	92	115	109	0.304
**Slope**		409	442	485	0.575	290	486	800	143	319	639	92	667	570	**0.000**
**Ethyl hexanoate**															
**Imax**	**1**	9729	13392	10746	0.379	7474	13370	15306	5492	2455	19308	2944	8622	26630	**0.002**
	**2**	1900	2200	2033	0.879	1805	1812	1162	404	2222	3832	287	2656	4220	**0.029**
	**3**	1000	1121	1186	0.910	704	927	730	217	911	2286	150	1823	2172	0.131
	**4**	387	508	573	0.491	340	295	758	111	226	952	122	714	884	**0.040**
**AUC**	**1**	324890	350894	319831	0.899	249348	342337	396553	132583	99899	713360	85345	325216	642204	**0.002**
	**2**	71289	74428	64905	0.740	69072	68814	77419	20990	66530	122149	9946	96815	100130	**0.004**
	**3**	37426	43700	45030	0.768	38869	38809	52660	11844	32935	73726	7345	63770	58511	0.070
	**4**	17952	25106	24864	0.371	21289	18863	38532	6274	12017	35458	3878	39251	28207	**0.015**
	**Total**	451557	494128	454630	0.881	378577	468823	565164	171691	211381	944693	106514	525052	829052	**0.002**
**% of release**	**1**	69	69	71	0.642	66	74	68	77	49	75	80	60	78	**0.000**
	**2**	18	16	15	0.142	19	14	15	12	31	13	9	21	12	**< 0.001**
	**3**	9	10	9	0.907	10	8	10	7	15	8	7	12	7	**0.045**
	**4**	4	5	5	0.145	6	4	7	4	5	4	4	7	3	**0.009**
		**Wine type**				**Interindividual differences**									
	**Time (min)**	**WC**	**WE**	**WP**	**p-value**	**S1**	**S2**	**S3**	**S4**	**S5**	**S6**	**S7**	**S8**	**S9**	**p-value**
**T25**		4	4	5	0.673	4	3	5	5	3	5	4	3	6	0.617
**T50**		15	14	15	0.981	22	9	16	13	5	29	9	10	19	0.120
**T75**		38	36	42	0.459	52	37	46	31	20	46	37	36	42	0.059
**T80**		44	44	50	0.422	68	42	53	38	30	51	41	43	47	**0.034**
**T90**		83	79	86	0.834	104	73	102	65	71	103	48	91	87	0.158
**T95**		126	129	136	0.662	148	120	154	111	124	156	93	135	131	0.156
**Slope**		802	873	834	0.849	716	819	1185	331	673	1315	168	1266	1051	**0.001**
**Ethyl decanoate**															
**Imax**	**1**	514	673	643	0.103	558	675	724	472	557	868	147	634	855	**0.002**
	**2**	183	209	208	0.212	229	194	242	161	175	302	65	207	227	**< 0.001**
	**3**	133	138	151	0.516	165	127	178	108	130	230	47	140	142	**0.002**
	**4**	99	114	127	0.104	128	99	149	88	95	170	45	137	109	**0.001**
**AUC**	**1**	95615	138813	134301	**0.029**	118055	134755	156658	97361	108643	194947	20818	121877	153069	**0.001**
	**2**	35368	45831	45110	0.070	48545	40073	58200	30545	37745	69284	6638	40597	47300	**0.000**
	**3**	22493	26647	28736	0.216	30644	23409	38029	17122	21903	45631	3976	25840	27074	**0.000**
	**4**	14216	18523	20567	**0.041**	21014	14456	28483	10834	12909	30777	2859	21116	17470	**0.000**
	**Total**	167691	229814	228714	**0.031**	218257	212693	281370	155863	181199	340640	34292	209430	244913	**0.000**
**% of release**	**1**	58	60	59	0.501	55	63	55	61	59	57	62	58	63	0.183
	**2**	21	20	20	0.685	22	19	21	20	21	20	18	20	19	0.448
	**3**	13	12	13	0.383	14	11	14	12	13	13	11	12	11	0.530
	**4**	8	8	9	0.321	9	7	10	8	7	9	8	10	7	0.081
**T25**		13	12	12	0.419	13	11	14	11	12	14	13	11	11	0.091
**T50**		27	24	24	0.336	27	20	29	24	20	32	25	23	25	0.157
**T75**		61	56	58	0.520	69	48	67	53	44	73	55	59	57	0.080
**T80**		76	69	72	0.501	86	59	83	65	55	90	67	74	70	0.059

**T90**		128	116	123	0.365	138	105	139	114	101	146	118	122	117	0.082
**T95**		173	163	171	0.367	180	155	183	162	155	185	170	166	164	0.236
**Slope**		394	501	523	0.117	511	465	678	339	454	742	72	535	460	**0.001**
**Linalool**															
**Imax**	**1**	63	68	67	0.412	63	65	73	62	63	77	59	61	71	0.140
	**2**	62	60	57	0.368	56	60	64	59	57	58	55	66	62	0.530
	**3**	56	55	55	0.978	53	51	60	52	53	58	53	61	58	0.302
	**4**	54	56	55	0.607	60	53	59	51	60	60	46	57	49	**0.045**
**AUC**	**1**	2478	2668	2761	0.480	1500	4192	3734	1714	495	4383	1056	2429	3743	**< 0.001**
	**2**	1957	1528	1670	0.242	747	2683	1694	786	784	2358	-	1893	1727	**0.004**
	**3**	1360	1353	1333	0.990	692	1599	2113	421		1727	-	1555	1274	**0.016**
	**4**	912	1075	1204	0.265	439	1158	1616		1315	1241	-	1260	885	**0.031**
	**Total**	7197	6105	7323	0.367	3378	9631	7732	3092	3054	9710	-	6601	7628	**0.006**
**% of release**	**1**	38	51	50	0.209	43	44	59	75	33	45	7	39	49	0.064
	**2**	27	22	21	0.028	22	28	21	28	22	24	12	31	22	**0.005**
	**3**	34	25	25	0.626	22	17	22	49	39	18	49	23	17	0.567
	**4**	25	36	28	0.568	13	12	16	56	48	13	89	16	11	**0.012**

		**Wine type**				**Interindividual differences**									
	**Time (min)**	**WC**	**WE**	**WP**	**p-value**	**S1**	**S2**	**S3**	**S4**	**S5**	**S6**	**S7**	**S8**	**S9**	**p-value**
**T25**		23	22	23	0.987	19	33	29	14	5	34	14	26	28	**0.014**
**T50**		47	54	53	0.495	56	69	64	34	13	68	32	72	56	**0.002**
**T75**		97	105	105	0.720	130	130	128	62	33	133	51	134	119	**0.001**
**T80**		108	121	122	0.487	149	146	141	76	41	154	59	151	134	**0.001**
**T90**		134	160	156	0.258	182	184	180	102	64	192	77	188	179	**0.001**
**T95**		153	183	175	0.217	203	210	204	120	72	217	92	205	209	**0.000**
**Slope**		16	19	19	0.469	12	24	30	7	9	26	7	29	20	**0.006**
**β-ionone**															
**Imax**	**1**	138	150	157	0.307	106	193	224	130	101	227	48	108	201	**< 0.001**
	**2**	119	109	125	0.165	86	156	167	100	88	173	41	99	150	**< 0.001**
	**3**	97	91	100	0.307	71	119	136	85	74	127	39	96	118	**< 0.001**
	**4**	83	79	91	0.088	66	101	119	67	66	111	41	88	101	**< 0.001**
**AUC**	**1**	27667	29761	33168	0.307	17467	44962	53765	22506	15825	53067	3000	15207	45990	**< 0.001**
	**2**	20709	19407	22546	0.288	10992	32106	35199	14664	10076	36966	1721	13995	32268	**< 0.001**
	**3**	14861	14278	16079	0.626	7351	22196	25615	8827	7232	25213	953	12056	21768	**< 0.001**
	**4**	12261	10698	12818	0.361	5242	16875	22325	6032	4646	17801	1261	9276	17060	**< 0.001**
	**Total**	74135	71206	84612	0.210	41052	116140	136903	52029	37779	133047	5287	50534	117086	**< 0.001**
**% of release**	**1**	41	49	39	0.268	45	38	40	42	41	40	72	30	39	0.112
	**2**	29	28	27	**0.014**	28	28	26	29	27	28	34	28	28	**< 0.001**
	**3**	18	18	19	0.658	17	19	18	17	19	19	11	24	19	**0.039**
	**4**	14	14	15	0.585	11	15	16	12	13	13	11	18	15	0.212
**T25**		48	46	50	0.494	43	58	55	48	39	55	29	51	54	**0.005**
**T50**		88	85	90	0.539	80	98	94	86	79	95	58	103	95	**0.005**
**T75**		146	139	148	0.391	134	155	155	139	145	151	106	161	152	**0.005**
**T80**		159	153	162	0.369	148	169	169	153	160	166	117	174	166	**0.005**
**T90**		187	187	199	0.277	181	201	201	191	195	199	149	204	200	**0.029**
**T95**		205	208	217	0.312	202	220	220	213	216	218	165	219	219	**0.028**
**Slope**		217	180	234	0.153	108	311	396	122	105	349	15	180	307	**< 0.001**

Imax: maximal intensity; **AUC:** area under the curve; **T25**: time to reach the 25% of total release; **T50:** time to reach the 50% of total release; **T75**: time to reach the 75% of total release; **T80:** time to reach the 80% of total release; **T90:** time to reach the 90% of total release; **T95**: time to reach the 95% of total release.
